# Characterization of bone only metastasis patients with respect to tumor subtypes

**DOI:** 10.1038/s41523-018-0054-x

**Published:** 2018-01-25

**Authors:** Amanda Parkes, Katherine Clifton, Aydah Al-Awadhi, Oluchi Oke, Carla L. Warneke, Jennifer K. Litton, Gabriel N. Hortobagyi

**Affiliations:** 1 Department of Cancer Medicine, The University of Texas MD Anderson Cancer Center, Houston, TX 77030 USA; 2 Department of Biostatistics, The University of Texas MD Anderson Cancer Center, Houston, TX 77030 USA; 3 Department of Breast Medical Oncology, The University of Texas MD Anderson Cancer Center, Houston, TX 77030 USA

## Abstract

Metastatic breast cancer (MBC) patients with bone only metastasis (BOM) are a unique population with limited characterization. We identified patients followed at MD Anderson Cancer Center from 01/01/1997 to 12/31/2015 for at least 6 months with a BOM diagnosis as first site of metastasis. Tumor subtype (TS) was assessed by initial breast biopsy immunohistochemistry using hormonal receptor (HR) and HER2 status, with four subtypes identified: HR+/HER2−, HR+/HER2+, HR−/HER2−, HR−/HER2+. HR+ was defined as estrogen receptor or progesterone receptor ≥1%. We identified 1445 patients with BOM, 1048 with TS data available. Among these patients, the majority were HR+/HER2− (78%). Median time from breast cancer diagnosis to first bone metastasis was 2.3 years (95% CI 2.1, 2.5) and varied significantly by TS, with longer time to distant disease in HR+/HER2− patients relative to all other TS (*p* < .0001). Median overall survival (OS) from breast cancer diagnosis was 8.7 years (95% CI 8.0, 9.7) and varied significantly by TS with poorer OS for HR−/HER2− and HR-/HER2+ patients relative to HR+/HER2− TS (*p* < .0001). The 442 patients with de novo BOM disease, defined as bone metastasis diagnosis within 4 months of breast cancer diagnosis, had significantly shorter OS (*p* < .0001). Overall, several higher risk BOM subsets were identified in this analysis, most notably HR−/HER2+ and HR−/HER2− TS and de novo BOM patients.

## Metastatic disease: Tumor subtype impacts outcomes for women with bone-only spread

Not all cases of breast cancer with bone-only metastases should be treated the same. Amanda Parkes and colleagues from The University of Texas MD Anderson Cancer Center in Houston, USA, examined the tumor subtype of over 1000 women with metastatic breast cancer that had spread to the bone. The majority—78%—tested positive for a hormone receptor (estrogen or progesterone) but negative for the HER2 receptor. The median time from diagnosis to identification of the first bone metastasis was 2.3 years and the median survival time post-diagnosis was 8.7 years, although this varied significantly by tumor subtype. Women negative for a hormone receptor or who developed bone metastases within 4 months of breast cancer diagnosis had the worst outcomes. The findings show the need to consider tumor subtype for this patient population.

## Introduction

Development of metastases in breast cancer patients constitutes the single largest risk factor for increased morbidity and mortality, with approximately 90% of deaths during treatment attributed to metastasis.^1,2^ Despite advances in breast cancer treatment, 13–30% of early breast cancer patients will develop distant metastases.^1,3^ Bone is the most frequent site of breast cancer metastasis, with bone metastases noted in 60–80% of metastatic breast cancer (MBC) patients, and is the first site of metastatic disease in 25–40% of MBC patients.^4,5^

Patients with bone only metastasis (BOM) are a unique MBC subpopulation, representing up to 51% of patients with bone relapse.^2^ Despite representing a significant number of MBC patients, these patients have routinely been excluded from clinical trials given bone only metastases have been defined as non-measurable per Response Evaluation Criteria in Solid Tumors (RESIST) criteria. The updated RECIST 1.1 criteria now include bone metastases with soft tissue masses greater than 10 mm as measurable disease, however this still excludes most patients with BOM. Despite representing a significant number of MBC patients, BOM patients are still inadequately characterized, limiting therapeutic strategies including clinical trial involvement.

Notably, studies to date characterizing BOM patients have been limited and of small sample size. There has also been limited evaluation of BOM patients with regards to breast cancer subtype, which is known to have prognostic significance and association with development of BOM.^6,7^ To further characterize this distinct MBC subgroup, we sought to describe the clinical characteristics of the largest sample of BOM patients thus far reported. Given the growing knowledge of the distinct clinical course and prognosis associated with tumor subtypes, we sought to characterize BOM patients by tumor subtype (TS) using hormonal receptor (HR) and HER2 status. We specifically sought to determine how TS affected outcomes in BOM patients.

## Results

Of the 2543 patients identified by the prospective database, 1098 were excluded for failure to meet inclusion criteria. Of these patients, 82 were found to have a coexisting malignant neoplasm, 172 had evidence of non-bone metastases at time of MBC diagnosis, 163 had a single bone metastasis at diagnosis without confirmatory bone biopsy, 654 did not have at least 6 months of follow up at MD Anderson Cancer Center, and 27 did not have a documented bone metastasis at time of MBC diagnosis. A total of 1445 patients met inclusion criteria and were evaluated for our study.

Of the 1445 BOM patients meeting inclusion criteria, the median age at time of breast cancer diagnosis was 49.3 years (range 20–94 years). Median age at diagnosis of BOM was 53.5 years (range 21–95 years). The baseline clinical characteristics are summarized in Table 1.

**Table 1:** Baseline clinical characteristics at time of BOM diagnosis of the 1445 patients meeting inclusion criteria, including sex, number, location, and type of bone metastases, and pain attributed to bone metastases as identified by physician review of the patient’s medical record

Characteristic	Number	Percent (%)
*Sex*		
Female	1432	99
Male	13	1
*Number of bone metastases at BOM diagnosis*		
Single	290	20
Multiple	1141	79
Unknown	14	1
*Location of bone metastases at BOM diagnosis*		
Axial skeleton	511	35
Appendicular skeleton	153	11
Axial + appendicular skeleton	770	53
Unknown	11	1
*Pain attributed to bone metastases at BOM diagnosis*		
Yes	909	63
No	329	23
Unknown	207	14
*Type of bone metastases at BOM diagnosis*		
Lytic	389	27
Sclerotic/Blastic	270	19
Mixed	149	10
Unknown	637	44

TS was assessed by initial breast biopsy immunohistochemistry (IHC) with the following four subtypes identified: HR+/HER2−, HR+/HER2+, HR−/HER2−, and HR−/HER2+. A total of 1052 patients had HR and HER2 characterization in our prospective database, which quantified estrogen receptor (ER) or progesterone receptor (PR) positivity as ≥1%, allowing for TS grouping. Notably, four of these patients had bilateral breast cancers with discordant TS between the bilateral breast biopsies and were therefore excluded from TS analyses. Ultimately, a total of 1048 patients met inclusion criteria and were included in TS analyses.

### Patient characteristics

Of the 1048 patients with TS available, the majority were HR+/HER2− (820 patients, 78%), while 11% (119 patients) were HR+/HER2+, 7% (76 patients) were HR−/HER2−, and 3% (33 patients) were HR−/HER2+.

As seen in Table 2, TS was significantly associated with age, race, and use of adjuvant hormonal therapy. Specifically, patients with HR+/HER2+ tumors were significantly younger than those with HR+/HER2− and HR−/HER2− tumors, and there was a higher percentage of HR+/HER2− tumors seen in non-Hispanic, white patients. As expected based on the receptor status associated with each TS, patients with HR+/HER2− tumors were most likely to receive adjuvant hormonal therapy, while patients with HR−/HER2− or HR−/HER2+ tumors were least likely to receive adjuvant hormonal therapy. TS subgroups did not differ significantly by bone metastasis location, number of sites of bone metastases, type of bone metastases, use of neoadjuvant or adjuvant chemotherapy, or use of bisphosphonate therapy. Notably, clinical stage at time of breast cancer diagnosis was documented for 65% (*n* = 684) of patients and close to half of patients in each TS subgroup were diagnosed initially with stage IV disease with no significant difference in de novo BOM diagnosis between TS subgroups. There was a trend toward statistically significance, however, with lower percentages of de novo BOM in the two HER2- subgroups (*p* = .096).

**Table 2:** Clinical characteristics stratified by TS, as assessed by initial breast biopsy IHC using HR and HER2 status, of 1048 BOM patients with TS available

Characteristic	HR+/ HER2−	HR+/HER2+	HR−/ HER2−	HR−/ HER2+	Total	*p*-value (4)
Median age at breast cancer diagnosis (years)	50.61	46.46	52.71	49.14	50.03	0.0074
(Minimum, maximum)	(23, 88)	(20, 83)	(27, 84)	(28, 70)	(20, 88)	
*Race/ethnicity n (%)*						
White, non-Hispanic	626 (76.34)	80 (67.23)	50 (65.79)	14 (42.42)	770 (73.47)	0.0004
Black, non-Hispanic	75 (9.15)	13 (10.92)	9 (11.84)	10 (30.30)	107 (10.21)	
Hispanic	89 (10.85)	16 (13.45)	13 (17.11)	7 (21.21)	125 (11.93)	
Asian/Pacific Islander	19 (2.32)	7 (5.88)	4 (5.26)	2 (6.06)	32 (3.05)	
Other	11 (1.34)	3 (2.52)	0 (0.00)	0 (0.00)	14 (1.34)	
*Bone metastasis location n (%)*						
Appendicular	93 (11.41)	10 (8.47)	8 (10.53)	3 (9.38)	114 (10.95)	0.1882
Axial	261 (32.02)	52 (44.07)	29 (38.16)	14 (43.75)	356 (34.20)	
Both axial and appendicular	461 (56.56)	56 (47.46)	39 (51.32)	15 (46.88)	571 (54.85)	
Number of bone metastases *n* (%)						
Single	153 (18.82)	23 (19.49)	15 (20.00)	8 (24.24)	199 (19.15)	0.884
Multiple	660 (81.18)	95 (80.51)	60 (80.00)	25 (75.76)	840 (80.85)	
*Type of bone metastasis at time of BOM diagnosis (1) n (%)*						
Blastic/Sclerotic	148 (30.33)	18 (29.03)	17 (39.53)	9 (52.94)	192 (31.48)	0.3101
Lytic	249 (51.02)	30 (48.39)	17 (39.53)	7 (41.18)	303 (49.67)	
Mixed	91 (18.65)	14 (22.58)	9 (20.93)	1 (5.88)	115 (18.85)	
*Neoadjuvant chemotherapy n (%)*						
No	640 (78.05)	94 (78.99)	61 (80.26)	28 (84.85)	823 (78.53)	0.7905
Yes	180 (21.95)	25 (21.01)	15 (19.74)	5 (15.15)	225 (21.47)	
*Adjuvant chemotherapy n (%)*						
No	518 (63.17)	67 (56.30)	40 (52.63)	20 (60.61)	645 (61.55)	0.1822
Yes	302 (36.83)	52 (43.70)	36 (47.37)	13 (39.39)	403 (38.45)	
*Adjuvant hormonal therapy n (%)*						
No	419 (51.10)	75 (63.03)	68 (89.47)	32 (96.97)	594 (56.68)	<0.0001
Yes	401 (48.90)	44 (36.97)	8 (10.53)	1 (3.03)	454 (43.32)	
*Bisphosphonate therapy after diagnosis of bone metastasis (2) n (%)*						
No	51 (7.04)	10 (9.71)	8 (14.04)	2 (7.14)	71 (7.79)	0.2405
Yes	673 (92.96)	93 (90.29)	49 (85.96)	26 (92.86)	841 (92.21)	
*Clinical stage at time of breast cancer diagnosis (3) n (%)*						
Stage I	48 (9.04)	3 (3.66)	4 (8.70)	1 (4.00)	56 (8.19)	0.4535
Stage II	130 (24.48)	22 (26.83)	12 (26.09)	8 (32.00)	172 (25.15)	
Stage III	96 (18.08)	12 (14.63)	10 (21.74)	1 (4.00)	119 (17.40)	
Stage IV	257 (48.40)	45 (54.88)	20 (43.48)	15 (60.00)	337 (49.27)	
*Bone metastases within 4 months of breast cancer diagnosis n (%)*						
No	529 (64.51)	67 (56.30)	53 (69.74)	17 (51.52)	666 (63.55)	0.096
Yes	291 (35.49)	52 (43.70)	23 (30.26)	16 (48.48)	382 (36.45)	

*N/A* not available

(1) Type of bone metastasis was unknown for 42%

(2) Use of bisphosphonate therapy after diagnosis of bone metastasis was unknown for 13%

(3) Clinical stage at time of breast cancer diagnosis was unknown for 35%

(4) *p*-values were derived from the *χ*^2^ test with the exception of age by tumor subtype which was tested using the Kruskal–Wallis test

### Time from breast cancer diagnosis to first bone metastasis diagnosis

**Fig. 1:**
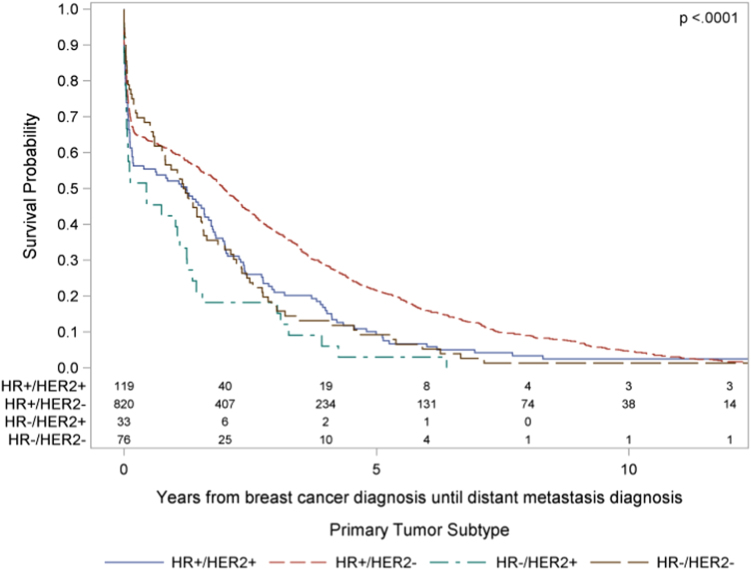
Time from breast cancer diagnosis to BOM diagnosis in years stratified by TS with pair-wise comparisons utilized to compare TS

The median time from breast cancer diagnosis to first bone metastasis diagnosis among the 1445 study patients was 2.3 years (95% CI 2.1, 2.5) with a range from 0.0 to 36.6 years. The time from breast cancer diagnosis to BOM diagnosis varied by TS (log-rank test, *χ*^2^(3) = 30.74, *p* < .0001), with pair-wise comparisons showing significantly longer time to distant disease in HR+/HER2− patients relative to all other TS. The HR+/HER2+, HR−/HER2−, and HR−/HER2+ TS did not differ significantly from each other in time from breast cancer diagnosis to BOM diagnosis (Fig. 1).

### Overall survival from breast cancer diagnosis

**Fig. 2:**
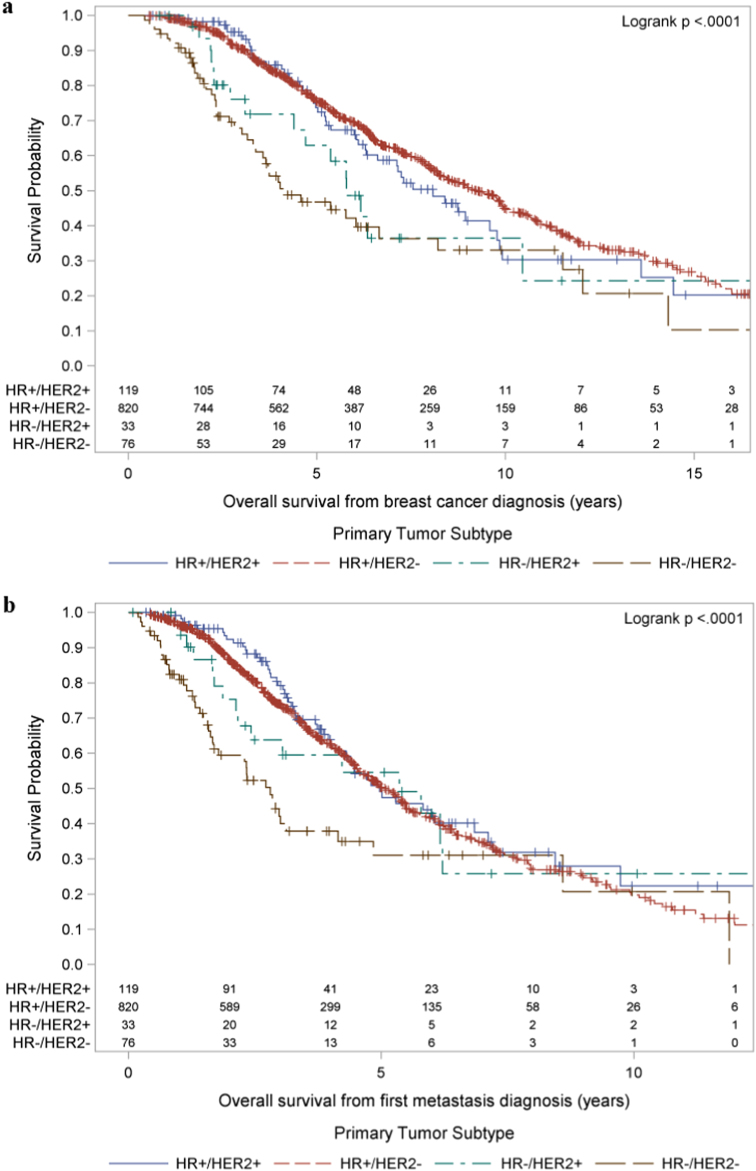
**a** OS from breast cancer diagnosis (top) and **b** OS from distant disease diagnosis (bottom), both stratified by TS

Among the 1048 patients with TS information available, there were 494 deaths observed during follow-up, and median follow-up among the 554 censored patients for OS was 5.6 years (range 0.6–26.2 years) from breast cancer diagnosis. Among the 1048 patients, the median OS from breast cancer diagnosis was 8.7 years (95% CI 8.0, 9.7). The OS varied significantly with TS (logrank test, *χ*^2^(3) = 26.80, *p* < .0001) with significantly poorer OS for HR−/HER2− and HR−/HER2+ as compared with HR+/HER2− TS (Fig. 2).

### Overall survival from distant disease diagnosis

Among the 1048 patients with TS information available, there were 494 deaths observed during follow-up, and median follow-up among the 554 censored for OS was 3.0 years (range 0.1–16.1) from distant disease diagnosis. The median OS from distant disease diagnosis was 4.9 years (95% CI 4.5, 5.4), and OS varied across TS (logrank test *χ*^2^(3) = 22.34, *p* < .0001), with patients with HR−/HER2− TS having significantly poorer OS relative to all other TS (Fig. 2).

### De novo bone only metastasis diagnosis

**Fig. 3:**
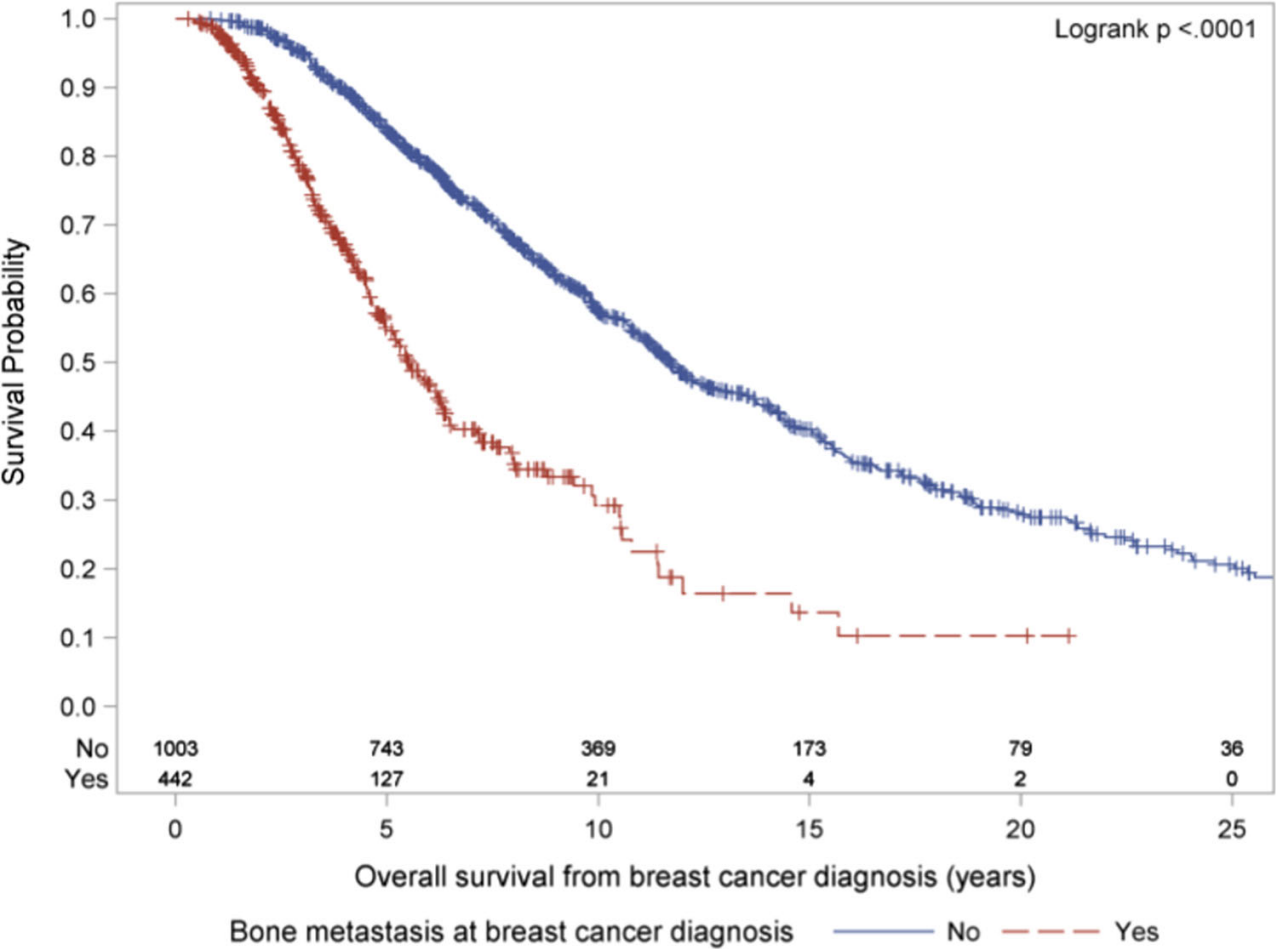
OS from breast cancer diagnosis in 442 patients with de novo BOM disease, defined as diagnosis of bone metastasis at the same time or within 4 months after breast cancer diagnosis

A total of 442 patients were diagnosed with bone metastasis at the same time or within 4 months of breast cancer diagnosis, defined as de novo BOM disease, and their OS from breast cancer diagnosis was significantly shorter than for those who were diagnosed with bone metastasis more than 4 months subsequent to breast cancer diagnosis, log-rank *χ*^2^(1) = 145.24, *p* < .0001. Median OS from breast cancer diagnosis among those with bone metastasis at breast cancer diagnosis was 5.5 years (95% CI 5.0, 6.3) compared to 11.7 years (95% CI 11.1, 12.7) among those who were diagnosed with bone metastasis more than 4 months subsequent to breast cancer diagnosis (Fig. 3).

### Hormone receptor positivity defined as ER or PR ≥ 10%

A secondary analysis was performed grouping TS based on HR+ defined as ER or PR positivity ≥10%. Of the 1048 patients included in initial TS analyses, 957 patients had precise quantification of ER or PR positivity available, allowing for differentiation of patients with HR+ defined as ER or PR ≥ 10%. There were no differences noted in the results of the above survival analyses when defining HR+ as ER or PR positivity ≥10%. Specifically, HR−/HER2− and HR−/HER2+ TS were found to have a significantly poorer OS from breast cancer diagnosis as compared to HR+/HER2− TS. OS from distant disease diagnosis was significantly poorer in HR−/HER2− TS patients relative to all other TS. Time from breast cancer diagnosis to BOM diagnosis was again found to be significantly longer in HR+/HER2− TS patients compared to all other subtypes when using the ER or PR ≥ 10% cutoff.

## Discussion

This study aimed to better characterize MBC patients with BOM, evaluating how discrete clinical features and TS affect outcomes in this distinct patient population. Given the number of BOM patients identified, this represents the single largest study to date of BOM patients. Unique clinical characteristics were seen in this BOM patient group, with the majority of patients at time of BOM diagnosis having multiple bone metastasis, most often lytic in nature and located in both the axial and appendicular skeleton, typically associated with pain.

With respect to TS classification, distinct clinical characteristics associated with TS grouping included a higher percentage of HR+/HER2− TS seen in non-Hispanic, white patients and significantly younger patients with HR+/HER2+ TS tumors as compared with HR+/HER2− and HR−/HER2− TS tumors. In concordance with earlier studies,^8^ BOM patients most commonly had HR+/HER2− TS. Median time from breast cancer diagnosis to first bone metastasis was significantly longer in patients with HR+/HER2− TS. Strikingly, we evaluated TS using two separate cut-offs to definite HR+, with no differences in survival outcomes using HR+ defined as ER or PR positivity ≥1 or 10%.

Notably, other studies have shown an improved prognosis for BOM patients as compared with MBC patients with visceral or central nervous system (CNS) metastases.^4,9–14^ Our study showed a median OS similar to that previously reported by Niikura et al. and Ahn et al. with median OS of 4.9 years from distant disease diagnosis and 8.7 years from breast cancer diagnosis.^15,16^ We showed poorer OS in HR−/HER2− and HR−/HER2+ TS, similar to a smaller study of 226 BOM patients by Diessner et al. which showed improved OS in luminal A patients as compared with basal like or HER2 patients with BOM.^17^

Interestingly, we found a much higher rate of de novo BOM disease at 30.6% (442 patients) than the typical 5–10% de novo rate seen in all MBC cases.^8^ When looking at these patients with de novo BOM disease, there was no statistically significant relationship to TS, but the OS from breast cancer diagnosis was significantly shorter than for those who were diagnosed with bone metastasis more than 4 months subsequent to breast cancer diagnosis.

Limitations of our study include the retrospective nature of our analysis and the use of IHC alone to define TS groupings. Additionally, our inclusion criteria of patients followed at MD Anderson Cancer Center from 01/01/1997 to 12/31/2015 for at least 6 months resulted in a more selected population. Our patient population did have a younger median age than the national average, likely reflecting the referral pattern to MD Anderson Cancer Center, but is a distinction that should not alter the conclusions of our analysis. Additionally, this referral pattern led to missing clinical stage at time of initial diagnosis in 35% of patients, as many of the patients first came to MD Anderson Cancer Center at the time they developed metastatic disease, which limited our ability to access the necessary information to stage the primary retrospectively. While an important distinction, again this should not alter the conclusions of our analysis. Given these aforementioned limitations, particularly the retrospective nature of this analysis using patients at a large referral center, we do not have sufficient data available to understand why only approximately 50% of patients with HR+/HER2− TS received adjuvant hormonal therapy, which we hypothesize could have been due to patient contraindications or refusal.

As the largest study to date of BOM patients, this study highlights that there are higher risk BOM subsets, most notably patients diagnosed with de novo BOM disease and those with HR−/HER2+ and HR−/HER2− TS. Given the substantial number of patients with BOM disease and the significantly poorer outcomes noted in some BOM patients, ongoing studies are warranted.

## Methods

### Patient selection

A prospectively maintained database of breast cancer patients was used to identify patients followed at MD Anderson Cancer Center from 01/01/1997 to 12/31/2015 for at least 6 months with a BOM diagnosis. We defined patients as having a diagnosis of BOM if bone was the only site of metastasis at the time of diagnosis with MBC. All BOM patients were included regardless of timing of bone metastasis diagnosis, however patients were classified as having de novo BOM disease if they were diagnosed with BOM within 4 months (112 days) of their initial diagnosis of breast cancer.^18^ Diagnosis of bone metastasis was confirmed with various modalities including computed tomography (CT), magnetic resonance imaging, positron emission tomography-CT, bone scintigraphy, and biopsy. Biopsy proven metastatic disease was required for inclusion in the study in patients with a single bony metastasis at time of MBC diagnosis. Patients with a coexisting malignant neoplastic diagnosis were excluded from the study. The Institutional Review Board approved the study; informed consent requirement was waived given the retrospective design of the study. The study was conducted in accordance with all relevant guidelines and procedures and approved by the ethical committee from the University of Texas MD Anderson Cancer Center

### Tumor subtype assessment and evaluation

TS was assessed by initial breast biopsy IHC with the following four subtypes identified: HR+/HER2−, HR+/HER2+, HR−/HER2−, and HR-/HER2+. HR+ was defined as ER or PR ≥ 1% by IHC. A separate analysis was also performed defining HR+ as ER or PR ≥ 10% by IHC. If strength of ER or PR positivity was not listed as a percentage, the following as noted on pathology reports were consider positivity ≥10%: at least 3+, “strongly positive”, “abundant”, “high positive”, “majority”, or “positive throughout most of the tumor”. If quantified as fm/mg cytosol protein in ligand-binding assays, a level of 10 fm/mg was considered equivalent to 10% ER or PR positivity.^19^ If the pathology report noted “weakly positive”, this was considered ER or PR 1–9% positive. With regards to HER2 characterization, IHC of 1+ was considered negative and IHC 3+ was considered positive, while IHC 2+ was inconclusive and relied on fluorescence in situ hybridization (FISH) results. If there was discordance in IHC and FISH results with regards to HER2, FISH was utilized. Patients with bilateral breast cancers were included in the study if there was concordance in TS. Four patients with bilateral breast cancers with discordant TS were excluded from TS evaluations.

### Bone metastasis characteristics

Bone metastasis characteristics were identified through physician review of the patient’s medical record. Sites of bone metastases, with division between the axial and appendicular skeleton, was identified at time of BOM diagnosis using clinician notes and imaging reports to identify location of bone metastases. As per their definition, bone metastases in the skull, ossicles of middle ear, hyoid, rib cage, sternum, and vertebral column were classified as axial skeleton metastases, while bone metastases in the pectoral girdle, arms, forearms, hands, pelvis, thighs, legs, feet, and ankle were classified as appendicular skeleton metastases. Also identified using clinician notes and radiologic reports was type of bone metastasis (blastic/sclerotic, lytic, or mixed) and number of bone metastases. We defined single bone metastasis as a solitary bone metastatic lesion restricted to a single site and multiple bone metastases as two or more lesions, including more than one lesion in the same bone. Mixed bone metastases were defined as the possession of two or more types of bone metastases in a patient at a single time point (such as a single radiologic report noting both lytic and blastic bone lesions in a patient). Pain at time of bone metastasis diagnosis was found through physician review of the patient’s medical record, utilizing documentation found in clinician notes.

### Treatment characteristics

We identified treatment characteristics for BOM patients, including receipt of neoadjuvant and adjuvant chemotherapy, hormonal therapy, and bone modifying agents. Bone modifying agents included bisphosphonates and denosumab.

### Last follow-up evaluation

Date of last follow-up was determined as either the date of death or the last patient contact noted in the electronic medical record, including both clinic visits and other forms of patient communication. At the time of last follow-up, all sites of metastatic disease, differentiating between visceral, bone, and CNS metastases, was determined through chart review of clinician notes and radiology reports.

### Statistical analysis

We summarized study sample characteristics using cross tabulations, frequencies, percents, medians, quartiles, and minimum and maximum values as appropriate. Associations with TS were analyzed using the *χ*^2^ test or Kruskal–Wallis test. We inspected variable distributions using histograms, boxplots, and the Ansari Bradley test, as appropriate. Time-to-event endpoints included OS from breast cancer diagnosis, OS from distant disease diagnosis, and time from breast cancer diagnosis to BOM diagnosis. Time-to-event endpoints were analyzed using the Kaplan–Meier method. For OS endpoints, patients who were alive at the end of follow-up were censored at the date of last follow-up. Equality across strata was tested using the logrank test. Time point estimates are presented with corresponding 95% confidence intervals that were obtained by applying the log–log transformation to the survivor function. For time-to-event endpoints, we adjusted pairwise comparisons between TS for multiple comparisons based on Tukey’s studentized range test. Two-tailed *p*-values <.05 were considered statistically significant, and all analyses were conducted using SAS for Windows (release 9.4, SAS Institute, Cary, North Carolina, USA).

### Data availability

The datasets gathered and analyzed during the current study are available from the corresponding author on reasonable request.
